# Written Emotional Disclosure Can Promote Athletes’ Mental Health and Performance Readiness During the COVID-19 Pandemic

**DOI:** 10.3389/fpsyg.2020.599925

**Published:** 2020-11-27

**Authors:** Paul A. Davis, Henrik Gustafsson, Nichola Callow, Tim Woodman

**Affiliations:** ^1^Department of Psychology, Umeå University, Umeå, Sweden; ^2^Department of Health Sciences, Karlstad University, Karlstad, Sweden; ^3^Norwegian School of Sport Sciences, Oslo, Norway; ^4^School of Sport, Health, and Exercise Sciences, Bangor University, Bangor, United Kingdom

**Keywords:** emotion, emotion regulation, elite athletes, sport psychology delivery, performance preparation

## Abstract

The widespread effects of the coronavirus disease 2019 (COVID-19) pandemic have negatively impacted upon many athletes’ mental health and increased reports of depression as well as symptoms of anxiety. Disruptions to training and competition schedules can induce athletes’ emotional distress, while concomitant government-imposed restrictions (e.g., social isolation, quarantines) reduce the availability of athletes’ social and emotional support. Written Emotional Disclosure (WED) has been used extensively in a variety of settings with diverse populations as a means to promote emotional processing. The expressive writing protocol has been used to a limited extent in the context of sport and predominantly in support of athletes’ emotional processing during injury rehabilitation. We propose that WED offers an evidence-based treatment that can promote athletes’ mental health and support their return to competition. Research exploring the efficacy of the expressive writing protocol highlights a number of theoretical models underpinning the positive effects of WED; we outline how each of these potential mechanisms can address the multidimensional complexity of the challenging circumstances arising from the COVID-19 pandemic (e.g., loss of earnings, returning to training and competition). Considerations and strategies for using WED to support athletes during the COVID-19 pandemic are presented.

## Introduction

The widespread effects of the coronavirus disease 2019 (COVID-19) pandemic have included a range of severe implications for sport competition and athletes. The cancellation of major events and the termination of competitive seasons have impacted athletes from the Olympic level to youth recreational participation (Nicola et al., 2020). It has been noted that the suspension of competition has been experienced by some athletes as a tragic loss (Schinke et al., 2020) inducing significant grief, frustration, and stress (Toresdahl and Asif, 2020). Moreover, as athletes are required to adapt to life in the context of a global pandemic, reports of the detrimental effects on mental health have mounted with increasing accounts of depression and symptoms of anxiety (Cao et al., 2020; Hull et al., 2020).

Imposed social isolation in the form of quarantine or preventative lockdowns is an unfamiliar and unpleasant experience; emotional distress may be amplified by inhibited availability of social support and preclusion of routines that might be acting as coping strategies (Usher et al., 2020; Van Bavel et al., 2020). Athletes in particular are accustomed to operating within extensively planned training and competition schedules that provide routine and structure to both sport-related interpersonal interactions as well as social recovery (Kellmann, 2010; Heidari et al., 2019). The severing of social support incurred as a result of an imposed quarantine or prescribed isolation can dissolve an individual’s sense of connectedness and impact negatively on mental health (Hawryluck et al., 2004; Williams et al., 2018). It has been highlighted that during the time of government-imposed restrictions (e.g., lockdown, social distancing) aimed at limiting the effects of the pandemic, individuals would benefit from maintaining social connections to minimize the negative impact on emotional well-being (Courtet et al., 2020).

One of the central functions of social support in sport is to provide athletes with the opportunity for emotional expression and associated emotional processing (Tamminen and Gaudreau, 2014). Restricted emotional processing has been linked with a range of negative health implications including elevations in sympathetic activity (Gross and Levenson, 1993), limiting of positive emotional experiences (Duddu et al., 2003), and a reduction of adaptive physiological responses (Clapp et al., 2015). One common technique to promote emotional processing is the emotional expression protocol of *Written Emotional Disclosure* (WED; Pennebaker, 1989). The expressive writing paradigm has been extensively researched in a wide variety of settings with diverse populations (for reviews, see Frisina et al., 2004; Frattaroli, 2006; Travagin et al., 2015; Riddle et al., 2016). There are, however, very few articles examining its applicability in the domain of sport, and these have predominantly focused on its use with injured athletes (e.g., Mankad et al., 2009a; Duncan et al., 2013; Salim and Wadey, 2018).

## WED: History and Application

Presented as a form of writing therapy for over 30 years, WED has been used as a method to reduce the inhibition of emotions through a systematic program of emotional expression (Pennebaker and Beall, 1986). In the vast majority of studies, the typical protocol requests participants to write expressively about an upsetting topic (e.g., previous trauma) for a prescribed duration of time, most often between 15 and 20 min, on three to five occasions over the period of a week (Frattaroli, 2006). A variety of positive psychological outcomes (e.g., reduced stress and anxiety; Lovell et al., 2016; Harvey et al., 2018) and physical benefits (e.g., immunological, lung function; Warner et al., 2006; O’Cleirigh et al., 2008) have been associated with completing the writing protocol.

Psycho-immunological benefits associated with completion of WED have been observed with athletes rehabilitating from long-term injury (Mankad et al., 2009b). In particular, emotionally expressive writing about the occurrence of the injury was associated with reduced stress levels and mood disturbance (e.g., confusion, depression) as well as enhanced expression of CD4+ immune cells. Taken collectively, Mankad and colleagues suggest that the potential psychophysiological benefits of WED may enhance immune health and mitigate the potential negative effects of emotional distress among rehabilitating athletes.

Clinical populations have also experienced positive outcomes from the protocol (Frisina et al., 2004). For example, patients with cancer (Stanton et al., 2002), post-traumatic stress disorder (PTSD) (Smyth et al., 2008), and asthma (Smith et al., 2015) have shown favorable responses to the treatment. Although, it is worth noting that physical benefits have been noted in a number of studies across a range of conditions (e.g., rheumatoid arthritis, chronic pelvic pain) in the absence of significant psychological improvement (e.g., Norman et al., 2004; Van Middendorp et al., 2009). Beyond medical conditions, expressive writing is highlighted as potentially beneficial for a wide range of populations including caregivers (Riddle et al., 2016), college students (Lumley and Provenzano, 2003), and individuals who are unemployed (Soper and Von Bergen, 2001) or suffering bereavement (Pennebaker et al., 1997; Lichtenthal and Cruess, 2010).

Studies using WED with injured athletes have proposed that the treatment’s therapeutic effects may be associated with grief-related responses to injury that can be attenuated when athletes gain greater personal understanding of their injury through emotionally expressive writing (Mankad and Gordon, 2010). Reports from athletes suggest that the COVID-19 cancellation of sport championships (e.g., 2020 Olympics) was experienced like a death (Schinke et al., 2020). The wider implications of discontinued competitive sport seasons were perceived by some athletes to have killed their career (Clarkson et al., 2020) as well as the development of their self-identity as an Olympian (Stephan and Brewer, 2007). WED is noted as an opportunity for individuals to process emotions induced by events occurring within social contexts (e.g., exclusion) that can impact upon self-identity (Cangià, 2014). That said, a number of individual difference factors (e.g., personality; Zakowski et al., 2011) have been highlighted to influence the effectiveness of the expressive writing protocol (Smyth, 1998). Further, research using WED with injured athletes notes that psychological and physical benefits can vary and may be associated with diverse participant characteristics and differing writing protocols (e.g., Duncan et al., 2013; Salim and Wadey, 2018). In consideration of using WED with athletes during the COVID-19 pandemic, examining the underpinning mechanisms of the intervention is warranted to evaluate its potential applicability and to optimize the possible psychophysiological outcomes (Smyth and Pennebaker, 2008).

## Mechanisms Underpinning WED

Research exploring the potential mechanisms underpinning the positive effects of WED has highlighted a number of theoretical models (Sloan and Marx, 2004; Frattaroli, 2006). Specifically, reductions in inhibition (Pennebaker and Beall, 1986; Pennebaker, 1989), positive cognitive adaptation (Margola et al., 2010), repeated exposure/emotional processing (Sloan et al., 2005), and increased social integration (Pennebaker and Graybeal, 2001) are proposed as possible explanations of the efficacy of the emotionally expressive writing protocol. However, despite extensive research, no single theoretical model is able to account fully for the effectiveness of WED (Pennebaker and Chung, 2007). In consideration of the multidimensional complexity of the challenging circumstances arising from the COVID-19 pandemic (e.g., disrupted training and competition schedules, reduced proximity of social support), WED could offer a particularly useful treatment due to its multiple potential mechanisms. Further, the multiple proposed mechanisms underlying the expressive writing protocol may vary in their prominence and influence across the dynamic conditions (e.g., quarantine, return to competition) that athletes are attempting to manage. Within the following sections, we will outline each of the proposed theoretical models underlying WED in relation to how they could address implications of the COVID-19 pandemic on athletes’ physical and mental health. We also offer guidance outlining how the potential psychological benefits associated with the proposed theoretical models may be maximized through recommendations for applied practice with athletes.

### Reductions in Emotion Inhibition

Emotional inhibition is characterized by an individual’s maladaptive interaction with his or her social environment and impacts upon physiological, cognitive, and social–behavioral processes (Traue et al., 2016). The first theoretical model that came to prominence in research attempting to account for the beneficial effects of WED had inhibition at its core (Sloan and Marx, 2004), and initial expressive writing treatment programs were guided by the Freudian concept of (dis)inhibition and its proposed influence on health (cf., Pennebaker and Beall, 1986; Pennebaker, 1989). Following the proposition that inhibiting thoughts, emotions, or behaviors are associated with unconscious physiological effort, the writing exercise provides an outlet to express the inhibited content and thus reduce the internal psychophysiological tension (Pennebaker and Chung, 2007). However, there is no evidence to support the position that decreases in inhibition mediate the relationship between expressive writing and health improvements (Sloan and Marx, 2004). Further, due to measurement difficulties inherent to the attempted examination of individuals’ inhibition of thoughts and emotions, little research has focused on this proposed theoretical model (Pennebaker and Chung, 2007).

Repression is a coping strategy that is closely associated with the inhibition of emotions and is operationally defined as the discrepancy between reported emotional experience and physiological indices of activation (Weinberger and Davidson, 1994). Research in sport highlights that self-reported levels of state anxiety do not differ between repressors and athletes low in trait anxiety (Mullen et al., 2009); however, when self-report data are compared with physiological indices of anxiety, athletes categorized as repressors (i.e., low self-report anxiety, high physiological reactivity) are found to suffer performance failures to a greater extent (Woodman and Davis, 2008).

*Inhibition* of information is an active, energy-consuming process (Schwartz and Kline, 1995). Conversely, *repression* involves no awareness of the concealment of emotions. As such, the repression of grief-related emotions induced by COVID-19 (e.g., cancellation of sport competition) may induce discrepancies between athletes’ overtly expressed emotions and their concomitant physiological state; any such perpetual emotional dissonance can promote the development of exhaustion (Kenworthy et al., 2014; Romo, 2017). More broadly, the inhibition of emotions associated with stress has a negative impact on health (Traue et al., 2016).

Another form of emotion inhibition that has been studied in athletes is *suppression* (Uphill et al., 2012; Wagstaff, 2014; Calverley et al., 2020). Whereas repression is unconscious, the emotion regulation strategy of expressive suppression involves conscious attempts to inhibit emotion-expressive behavior once the emotional state has already arisen in the individual (John and Gross, 2004). Suppression can reduce athletes’ behavioral expressions even though the psychophysiological symptoms of negative emotions are still experienced (Campbell-Sills et al., 2006). Research in the domain of sport highlights that emotion suppression may heighten awareness of negative emotions (Lane et al., 2012). Suppression also diverts cognitive resources toward attempts at emotion regulation, thereby reducing allocation invested in task-related demands and subsequently worsening performance (Wagstaff et al., 2013).

Research examining associations between the use of emotion regulation strategies and the psychological impacts of the COVID-19 pandemic highlights that reports of post-traumatic stress symptoms are positively related with expressive suppression and negatively associated with cognitive reappraisal (Jiang et al., 2020). These findings outline that inhibiting outward emotional expressions may amplify the experience of stress and negative emotion (Moore et al., 2008). In order to maximize the potential benefits of reductions in inhibition, it may be important to highlight the safety and non-judgmental context offered to athletes in the writing environment. Athletes who feel secure in their ability to authentically express their pandemic-related emotions (e.g., grief) through WED may reduce the negative effects of emotion suppression and in turn facilitate the cognitive reframing of their perceptions of the stressful situation.

### Cognitive Adaptation

The second theoretical model posited to explain the mechanisms that underpin WED is *cognitive adaptation* (Sloan and Marx, 2004). Central to cognitive adaptation is the processing of stressful events through the reworking of existing schemas. In particular, individuals must endeavor to redesign a conceptual system wherein the stressful events are assimilated into an established set of assumptions or alter the assumptions to accommodate the stress inducing event (Janoff-Bulman, 2010). Research suggests that translating a traumatic event into language allows an individual to organize, structure, and make sense of disturbing events that may not have been previously possible (Pennebaker and Francis, 1996; Pennebaker et al., 1997). According to this model, the process of gaining insight and cognitively assimilating emotional events into existing schemas decreases demands on working memory and reduces uncertainty regarding the stressful situation (Klein and Boals, 2001; Graybeal et al., 2002; Schwartz and Drotar, 2004). Making sense of COVID-19 measures (e.g., quarantine, canceled sporting events) is important for athletes’ positive stress adaptation and emotional well-being (Davis et al., 2019). Undertaking WED may encourage athletes to consider the implications of restrictions on sport participation in relation to wider global priorities (e.g., mortality rates) and promote their perspective taking, empathy, and mental health (Botterill and Brown, 2002; Meyer and Fletcher, 2007).

Expressive writing can facilitate participants’ re-evaluation of their role in the stressful situation and promote perspective taking that increases either the internalization or externalization of the events that transpire (Pennebaker et al., 2003; Moore et al., 2009). In support of this proposal, Campbell and Pennebaker (2003) found that participants who cognitively altered their individual and social perspectives over the course of the WED treatment were more likely to experience health benefits compared with participants with more rigid perspectives. More specifically, increased self-distancing (i.e., shifting from an internal viewpoint to an external observer perspective) has been found to mediate the association between expressive writing and reductions in emotional reactivity (Park et al., 2016).

Expressive writing can also reduce cognitive confusion surrounding an emotional event and provide an opportunity for the integration of antecedents and outcomes into an individual’s cognitive representation of themselves (Pennebaker and Francis, 1996; Graybeal et al., 2002). Hudson and Day (2012) note that athletes may re-evaluate and augment their perspective as a result of undertaking an expressive writing intervention when the protocol instructions align with reversal theory. Adapting writing instructions can promote individual growth by reducing avoidance of stressors, facilitating problem solving, and increasing reflection upon competition and training strategies.

The expressive writing protocol offers an avenue for structured self-reflection. Re-evaluating holistic life balance, re-setting of goals, and revisiting competition plans have been noted as being central for maintaining athletes’ mental health during the upheaval of COVID-19 restrictions (Schinke et al., 2020). WED presents an opportunity to independently increase self-awareness and integrate disconnected thoughts and feelings with aspects of identity (Brody and Park, 2004). Previous research examining the role of cognitive adaptation has typically analyzed participants’ written texts to assess changes in the use of pronouns and words reflecting insight (Fuentes et al., 2018). Computer programs that conduct linguistic analyses have enabled researchers to track changes in participants’ writing and link word usage with various health and behavioral outcomes (Pennebaker et al., 1997). Previous investigations of WED with injured athletes have highlighted changes in the semantics of language over repeated sessions of expressive writing, specifically, a significant increase in positive emotions and words related to cognitive mechanisms (e.g., because, therefore), as well as a decrease in negative emotion words (Salim and Wadey, 2018). Athletes attempting to cohere disconnected thoughts and fluctuating emotions during the COVID-19 pandemic may benefit from increased self-awareness, acceptance, and compassion (Coyne et al., 2020). In an effort to promote cognitive adaptation, we recommend adapting the instructions for each of the sessions across the program of writing to facilitate a process of reflection that develops athletes’ self-awareness, perspective taking, and compassion for themselves and others. Repeated sessions of expressive writing could improve athletes’ understanding of emotions, thoughts, and behavioral experiences in relation to their evolving identity during the pandemic.

### Exposure Hypothesis

Repeated emotional expression underpins WED’s third theoretical model of the *exposure hypothesis* (Frattaroli, 2006). Exposure-based therapies guide participants through repeated contact with aversive stimuli that triggers unpleasant emotions, while simultaneously engaging in a behavior that is incongruent with the induced affective state (Ljótsson et al., 2011). Modern forms of exposure have demonstrated high efficacy in many different anxiety disorders, including panic disorder, specific phobias, social anxiety, and obsessive–compulsive disorder (Buchholz et al., 2019). Through repeated exposure to the traumatic stimuli *via* the expressive writing program, participants experience a reduction in emotional arousal that results in the habituation of participants’ stress responses and leads to beneficial health outcomes (Kloss and Lisman, 2002; Sloan et al., 2005; Smyth et al., 2008). Sloan and Marx (2004) suggest that WED can serve as a less threatening context for participants to recall traumatic memories that they had previously avoided. Exposure is considered a core method in Cognitive Behavioral Therapy (CBT; Hedman et al., 2014), although it is seldom described in sport settings (Gustafsson and Lundqvist, 2020). Exposure in the form of expressive writing would likely serve as a useful method when exposure cannot be used *in vivo*; for example, in an effort to reduce an athlete’s stress associated with recommencing international air travel. It is anticipated that emotional distress may arise due to uncertainty around safety (e.g., risk of infection) or travel disruption (e.g., changes to quarantine requirements), and that undertaking systematic exposure through expressive writing may help to reduce psychophysiological stress reactivity and increase an athlete’s preparedness to travel. For example, athletes may be guided to write about effective emotion regulation strategies or tactics to promote positive stress adaptation to be used if a flight is canceled (Gärling et al., 2020). Rehearsed systematic exposure to emotions associated with flight cancellations (e.g., shock, anxiety) can facilitate more efficient emotional processing if/when they arise under pressure filled circumstances (e.g., crowded airports).

It is worth noting that WED incorporates many similar features of acceptance that comprise Acceptance Commitment Therapy (ACT; Hayes, 2004); underpinning both ACT and WED is the guidance for individuals to adopt a position of openness to all thoughts, feelings, and sensations that arise during the course of the treatment (Pennebaker, 1997; Gardner and Moore, 2010). The opposite attitude to acceptance is experiential avoidance; research outlines that experiential avoidance is associated with multiple negative outcomes, such as anxiety, depression, and lower quality of life (Hayes et al., 2004, 2006). Initial research also confirms related findings using ACT with injured athletes returning to competition (Shortway et al., 2018). Therefore, WED may serve to help athletes “open up” to experiences that are deemed uncomfortable and distressful; subsequent decreases in emotional avoidance could result in increased well-being.

In particular, the thought of returning to sport competition following the easing of COVID-19 restrictions is likely to induce intense emotions for many athletes. Uncertainty in terms of the risk of infection, as well as concerns over performance readiness and injury, may be sources of emotional distress for athletes (Mohr et al., 2020). Athletes completing the expressive writing program can be systematically exposed to the specific stressful stimuli associated with returning to competition and subsequently experience a reduction in emotional arousal that ultimately results in the habituation of participants’ stress responses.

The necessity to adapt the competition environment (e.g., no spectators) may present novel circumstances that result in athletes’ perceptions of reduced challenge and/or motivation; for example, athletes may be required to undertake matches in the absence of a potential audience effect (Wallace et al., 2005). As such, athletes may perceive a “lack of emotional demand” and experience a state of under arousal that is discordant with their individual zone of optimal functioning (Hanin, 1997). In particular, personality, and specifically narcissism, has been found to influence emotional responses to competition and moderate the emotion–performance relationship (Roberts et al., 2018). Narcissists may reduce their effort due to the lack of opportunity for glory in the absence of an audience and experience subsequent performance decrements (Wallace and Baumeister, 2002; Beattie et al., 2017). In an attempt to prepare for the lack of spectators, narcissists may benefit from engaging in external visual imagery as a strategy to vicariously simulate performance-enhancing emotional states (Roberts et al., 2010). Combining WED with imagery training (Lang et al., 1980, 1983) aimed at optimizing athletes’ psychophysiological readiness to return to competition may amplify the positive effects of both the expressive writing treatment and mental rehearsal (Konig et al., 2014).

The benefits of mental rehearsal and imagery in athletes’ preparation and performance are well documented (Martin et al., 1999; Slimani et al., 2016). Imagery is noted to be effective in developing the self-efficacy of athletes during injury rehabilitation and preparation for return to competition (Wesch et al., 2016). Research on imagery has shown that athletes who can imagine themselves performing successfully often experience enhanced performance outcomes (e.g., Callow et al., 2017, 2019). In particular, it is noted that athletes possessing a high task orientation are effective in forming mastery images and experience the emotion of these images (Gregg et al., 2016). On a related note, the benefits of WED have also been observed in studies that have adapted instructions directing participants to write expressively about life goals and best possible selves (King, 2001; Harrist et al., 2007; Layous et al., 2013). Directing athletes to write about their best possible return to sport may help focus their pursuit of this goal. Combining imagery and expressive writing associated with mastery goals, rather than achievement alone, may better prepare athletes for the emotional experience of returning to competition.

The combined use of constructing mental imagery and verbal representations, in the form of expressive writing, links with Paivio; Paivio’s (1971; 1986) dual-coding theory of information processing. Treatment of clinical anxiety disorders has been developed in consideration of the dual-coding theory (Martin, 1991; Stöber, 1998) with the aim of reducing worry by enhancing the concreteness of imagery and decreasing avoidance (Stöber and Borkovec, 2002). Athletes’ potential emotional distress and worry surrounding the COVID-19 pandemic may be reduced by improving the concreteness of imagery through the development of verbal representations in the language used in the expressive writing protocol. For example, worries about returning to competition may be reduced if athletes are better able to clearly articulate the image of successfully completing tasks associated with recommencing sport (e.g., simultaneously writing about and visualizing their first physical contact with a player on the opposing team).

As athletes engage in a process of identifying process goals directed toward a successful return to sport competition, it is likely that they will seek advice from associated support teams (e.g., coaches; Arthur et al., 2019). As a result of increasing social interactions, they may begin to express their emotions more widely in social contexts. It is noted that emotional expression undertaken in experimental laboratory writing protocols associates with changes in social interactions and language use in the real world (Pennebaker and Graybeal, 2001).

### Social Integration

The final proposed explanation for the efficacy of WED outlines that linguistic changes resulting from completion of the expressive writing protocol are more widely integrated into social interactions (Pennebaker and Graybeal, 2001). The theoretical model of social integration posits that WED can facilitate the organization and articulation of emotions that subsequently promotes wider sharing and reciprocal social support (Bryne-Davis et al., 2006). Athletes who undertake expressive writing independently within the WED paradigm may alter the manner in which they interact with their social world as a result of the previously discussed mechanisms underlying WED (i.e., reduced inhibition, cognitive adaptation, exposure). For example, athletes who were planning to attend the Olympics in Japan may have initially struggled with articulating and expressing their feelings of loss about the cancellation of the event. Over the course of repeatedly expressing their emotions systematically during the writing protocol, athletes’ emotional arousal may be reduced, and their thoughts about the setback could be better organized as a result of increased acceptance and self-compassion (Mosewich et al., 2013; Reis et al., 2015). Consequently, they may feel more comfortable speaking with family and more effective in communicating their support needs.

Disclosing thoughts, emotions, and personal experiences *via* written posts on social media has increased during the COVID-19 pandemic (Nabity-Grover et al., 2020). It has been noted that athletes’ presence on social media during the pandemic can serve as positive role models (Leng and Phua, 2020). Examples of athletes’ adapted training techniques have served to inspire physical activity during government-prescribed lockdowns (Hayes, 2020). Further, athletes can maintain a sense of connection and fan engagement *via* posts on social media (e.g., Su et al., 2020). Sharing emotional experiences as well as useful information with fans, friends, and family across social media interactions is noted as a potential means to regulate pandemic-related anxiety (Wiederhold, 2020). That said, social media use can contribute to mental health problems; frequent exposure during the COVID-19 pandemic is linked with greater reports of depression and anxiety (Gao et al., 2020). A pressure to present an ideal image of oneself is identified as a contributing factor of psychological distress associated with the use of social media (Casale et al., 2014; Keles et al., 2020). WED undertaken independently provides an opportunity for authentic emotional expression without fear of judgment from others, gaining self-compassion and acceptance through the writing paradigm may reduce anxieties related to idealized self-presentation on social media (Zhang et al., 2020).

Increased social integration and interactions arising as a result of WED have been evidenced in studies outlining that participants assigned to experimental disclosure were more likely than controls to talk about their traumatic experience in the weeks or months following disclosure (e.g., Kovac and Range, 2000). However, it is important to note that expressive writing results in greater health benefits for individuals who perceive that social support is available (Milbury et al., 2017). As such, before athletes initiate WED, it may be prudent to identify and/or establish a supportive social network. The availability of a social support network may promote social integration by offering athletes an outlet when they gauge themselves as being ready to more widely discuss emotional aspects of their experience of the COVID-19 pandemic.

It is possible that the influence of the social integration theory underlying WED is realized over a longer duration than the other proposed mechanisms. Specifically, linguistic changes occurring throughout the completion of the writing are likely to reflect cognitive processes developing across repeated sessions (Pennebaker and Graybeal, 2001). Further, athletes’ mental health, and specifically their perceptions of the topic of the expressive writing, will be influenced by ongoing social interactions as well as the feedback gained from significant others. Coming to terms with the implications of a stressful situation, such as a global pandemic, appears to be associated with perceptions and emotions about oneself in relation to others (Swann, 1997; Campbell and Pennebaker, 2003).

The novelty of initiating a program of expressive writing may also instigate athletes’ sharing of their experience of WED. Athlete peer-support groups have been established in an attempt to offer peer support during the COVID-19 pandemic; supplementing these online meetings with independent completion of the expressive writing protocol may focus discussion topics among athletes and lead to collective problem-solving as well as emotional support. Sport psychologist, in particular, may act as moderators in online group discussions to help guide athletes’ shared exploration of emotional experiences and promote social support.

## Summary and Considerations for Use With Athletes

In review of the multiple theories advanced in attempts to explain the underpinning mechanisms of WED, it is likely that multiple interacting factors are driving the effectiveness of expressive writing. Considering the wide-ranging implications of the COVID-19 pandemic, it may be particularly useful to implement an intervention to support athletes that can potentially impact upon a variety of aspects of stress (e.g., grieving, isolation, uncertainty). To this end, the writing paradigm appears to affect individuals along multiple dimensions (e.g., emotional, cognitive, physiological, social).

Although it is unclear which underlying mechanisms might be most impactful, the utility of the writing technique is clear given its adaptability across differing situations, participants, conditions, and settings. For example, the writing protocol has been observed to be effective even when the time of the writing session is dramatically reduced (i.e., 2 min; Burton and King, 2008). Shortening the length of the writing sessions (e.g., 5–10 min) may increase athletes’ receptivity to undertaking the protocol and minimize concern about time constraints. On a similar note, a single session of expressive writing for a duration of 10–15 min has been highlighted as being effective in the reduction of negative emotions when participants are guided to reframe thoughts and feelings using positive emotion words (Fernández and Páez, 2008). The concept of reframing negative thoughts to more positive alternatives is widely known by athletes who have undertaken mental skills training focusing on self-talk (Van Raalte et al., 2016). In particular, the link between self-talk, affective states, and performance is well established in sport psychology (e.g., Hardy et al., 2001; Latinjak et al., 2017); athletes may be more open to engaging with the expressive writing protocol if it is presented as a systematic organization of self-talk relating to their experience of the pandemic.

In terms of accessibility, the expressive writing protocol is ideally suited for use with athletes in quarantine and observing social distancing; many studies have highlighted WED’s effectiveness when undertaken at home or online (e.g., Gillis et al., 2006; Stockton et al., 2014). It is proposed that WED may be particularly helpful for individuals with limited availability of emotional outlets in their social environment (Zakowski et al., 2004). Although online platforms (e.g., Zoom) have been used extensively by coaches and sport organizations in attempts to bridge social distance during periods of lockdown, participants’ affective experience and perceptions of emotional expression can vary dramatically with diverse outcomes (Serrano-Puche, 2020).

During government-imposed restrictions, athletes may perceive a loss of autonomy and reduced locus of control. Undertaking WED may facilitate positive stress adaptation in response to socially prescribed behaviors; athletes may gain greater insight into how they can adopt a proactive role in responding to events rather than perceive themselves to be dictated to through restrictions that curtail usual training methods (Fallby et al., 2006; Holden et al., 2019). For example, WED can assist athletes in the evaluation of their daily routines and promote effective self-regulation in regard to the use of surplus time made available as a result of cancellations and altered schedules. Many athletes are used to maintaining training logs that record a range of factors and activities related to performance development (e.g., diet, sleep, training load); introducing WED as an extension of their training log may increase individual athletes’ uptake of the protocol.

It is worth noting that WED is not an ideal treatment for everyone, and that differential outcomes can be observed across varying characteristics of personality (e.g., alexithymia, neuroticism; Baikie, 2008; Zakowski et al., 2011). Sport psychologists would be well advised to ensure that there is adequate support for athletes in case WED initiates an intensity of emotional expression that the athlete feels inadequately prepared to manage. Further, WED can prompt a level of self-reflection that would benefit from the provision of an external perspective to assist with organizing thoughts associated with the uncertainty of the current situation and return to competition in the future. That said, a study by Salim et al. (2015) highlighted that injured athletes reporting low levels of hardiness perceived emotional disclosure as potentially being burdensome on others and could result in negative repercussions (e.g., team selection; Salim et al., 2015). Therefore, it is important for sport psychologists to first evaluate the suitability of WED for use with an athlete, in terms of both personality and quality of surrounding support. Moreover, athletes possessing limited motivation or ability to process their emotions through expression may find WED unappealing or counterproductive (Kraft et al., 2008). For example, athletes who struggle to identify or articulate the stressors they associate with the pandemic are less likely to experience cognitive or affective changes.

Caution is advised prior to commencing a program of WED, as writing about the events surrounding the pandemic (e.g., job loss, death of loved ones) can be an upsetting experience. Sport psychologist must ensure that the risk of potential retraumatization is managed effectively to realize the potential psychological benefits associated with undertaking the expressive writing program. Athletes may unduly suffer from WED if there is a failure to appropriately support the athlete during the experience of intense emotions associated with the writing or the potential secondary effects that could emerge from the sharing of thoughts and feelings with significant others (e.g., teammates).

To enhance the safety and efficacy of WED with athletes during the COVID-19 pandemic, future research investigating the use of the expressive writing treatment is warranted. In consideration of previous studies exploring WED across diverse populations and contexts, a variety of research designs (e.g., randomized control trials, qualitative interviews) and methods (e.g., measures of immunological functioning and emotional well-being) may help elucidate athletes’ experiences of WED. In particular, single-case research may facilitate understanding of the effectiveness of the expressive writing intervention and reflect dynamic aspects of an athlete’s emotional processing across multiple phases of the pandemic (e.g., initial cancellation of events, return to competition; Barker et al., 2013; Turner et al., 2020).

In summary, WED has been found to have health benefits across many populations including athletes. In consideration of the restrictions arising during the COVID-19 pandemic, the expressive writing protocol offers a flexible treatment that can be adapted to the individual athlete’s circumstances as well as supplement ongoing support. WED is an evidence-based treatment that can help maintain athletes’ mental health and support their return to competition. Research evaluating the actual psychological and physical health benefits of WED in athletes and coaches within the context of the COVID-19 pandemic is warranted, and related studies can help refine the expressive writing protocol to align with individual circumstances and optimize treatment outcomes.

## Author Contributions

All authors listed have made a substantial, direct and intellectual contribution to the work, and approved it for publication.

## Conflict of Interest

The authors declare that the research was conducted in the absence of any commercial or financial relationships that could be construed as a potential conflict of interest.
